# Androgen and Androgen Receptors as Regulators of Monocyte and Macrophage Biology in the Healthy and Diseased Lung

**DOI:** 10.3389/fimmu.2020.01698

**Published:** 2020-08-07

**Authors:** Mireya Becerra-Diaz, Mason Song, Nicola Heller

**Affiliations:** Anesthesiology and Critical Care Medicine, Johns Hopkins University, Baltimore, MD, United States

**Keywords:** androgen, androgen receptor, monocyte, macrophage, asthma, lung, sex difference, sex hormone

## Abstract

Androgens, the predominant male sex hormones, drive the development and maintenance of male characteristics by binding to androgen receptor (AR). As androgens are systemically distributed throughout the whole organism, they affect many tissues and cell types in addition to those in male sexual organs. It is now clear that the immune system is a target of androgen action. In the lungs, many immune cells express ARs and are responsive to androgens. In this review, we describe the effects of androgens and ARs on lung myeloid immune cells—monocytes and macrophages—as they relate to health and disease. In particular, we highlight the effect of androgens on lung diseases, such as asthma, chronic obstructive pulmonary disease and lung fibrosis. We also discuss the therapeutic use of androgens and how circulating androgens correlate with lung disease. In addition to human studies, we also discuss how mouse models have helped to uncover the effect of androgens on monocytes and macrophages in lung disease. Although the role of estrogen and other female hormones has been broadly analyzed in the literature, we focus on the new perspectives of androgens as modulators of the immune system that target myeloid cells during lung inflammation.

## Introduction

The immune system is essential for maintaining homeostasis within tissues and organs and protecting them against threats, such as harmful pathogens or cancerous transformation (1). It comprises both innate and adaptive components. The innate immune system is made up of the innate lymphoid (innate lymphoid cells [ILCs], natural killer cells [NKs], and lymphoid tissue inducers [LTi]) and innate myeloid subsets (2, 3). The innate immune system consists of a network of immune cells and molecules that provide rapid, first-line defense against pathogens. In contrast, the adaptive immune response, made up of B and T lymphocytes (4), takes days or even weeks to become established (5). Innate immune cells express pattern recognition receptors that recognize unique and conserved pathogen-associated molecular patterns such as lipopolysaccharide (LPS), viral ssRNA, and fungal β-glucan (6). B and T cells have evolved to recognize a finer repertoire of self- and nonself-antigens that facilitate pathogen-specific actions, immunologic memory generation, and host immune homeostasis regulation (4). To accomplish this, the adaptive immune response involves a tightly regulated interplay between T and B lymphocytes and antigen-presenting cells of the myeloid lineage, such as dendritic cells (DCs), monocytes, and macrophages (4). Myeloid cells arise from the bone marrow. The type and magnitude of the immune response is influenced by biological sex and age (7), and therefore differs between males and females. Sex differences in the function of the immune system arise from both genetic (chromosomal) sex differences and differences mediated by the action of male and female sex hormones. Because the concentration of sex hormones changes over the lifespan and throughout the course of the menstrual cycle in women, the function of the immune system also changes during different stages of life. Innate myeloid immune cells, like other cell types, express sex hormone receptors and are responsive to sex hormones (8).

Sex hormones are synthesized from cholesterol through a defined enzymatic cascade, predominately in the gonads and the adrenal glands (9). Sex hormones are also produced in other tissues, including the brain, placenta, mammary glands, liver, and adipose tissue (9–11). In addition to driving sexual development of egg and sperm production, sex hormones are responsible for the development of male and female secondary sexual characteristics, like breast development and growth of facial hair, that occur during puberty (12). Androgens include testosterone, dihydrotestosterone (DHT), androstenedione, androstenediol, and dehydroepiandrosterone (DHEA), with DHT being the most potent (13). The concentration of androgens in circulation is about seven-fold higher in adult men than in adult women (14, 15). Estradiol and progesterone are the predominant female sex hormones (16) synthesized by the ovaries and adrenal glands. Both male and female sex hormones are bound to the plasma proteins, albumin and sex hormone binding globulin (SHBG), and only a small percentage exists as free hormone (1–2%). Thus, the bioavailability of sex hormones is regulated by their biosynthesis and also the amount of albumin and SHBG.

Importantly, sex hormones mediate not only anatomic differences between women and men but also direct sex differences in immune responses, leading to different risks for immunologic diseases (17). Overall, women have a greater risk for autoimmune diseases (such as systemic sclerosis and systemic lupus erythematosus) (18), whereas men are more likely to die of infectious and parasitic diseases (19). Moreover, men have a greater risk of non-reproductive cancers (20–22). Both gender and sex are important mediators of these and other health and disease differences observed between men and women. While gender refers to the array of socially constructed roles, attitudes, personality traits, and behaviors, sex represents a biological characteristic of an individual (23), including the hormonal milieu and chromosome complement (22). In general, estrogens are considered to have proinflammatory properties and androgens are thought to have anti-inflammatory properties (24). In the United States (25) and worldwide (26), relevant evidence highlights important epidemiologic sex differences in incidence, susceptibility, and severity of a number of diseases that affect the respiratory tract. In this review, we will focus on how male sex hormones, the androgens, modulate the response of myeloid cells in the lung and how this modulation impacts the outcome of different diseases of the lung.

## Sex Differences in Human Lung and Lung Diseases

Biological sex mediates differences in the incidence and pathophysiology of lung diseases. These differences arise from sex differences in the structure and function of the lung itself, and also in the immune cells that populate the lung and are recruited to it during inflammation. Before birth, the female lung has several structural advantages over the male lung. Surfactant is produced earlier, and, although the female lung is smaller, it has more alveoli per unit area. Neonatal females have higher expiratory flow rates than do male neonates when corrected for size. Thus, male sex is a major risk factor for the development of respiratory distress syndrome, bronchopulmonary dysplasia in neonates (27–30), and asthma in childhood (31, 32).

**Figure 1:**
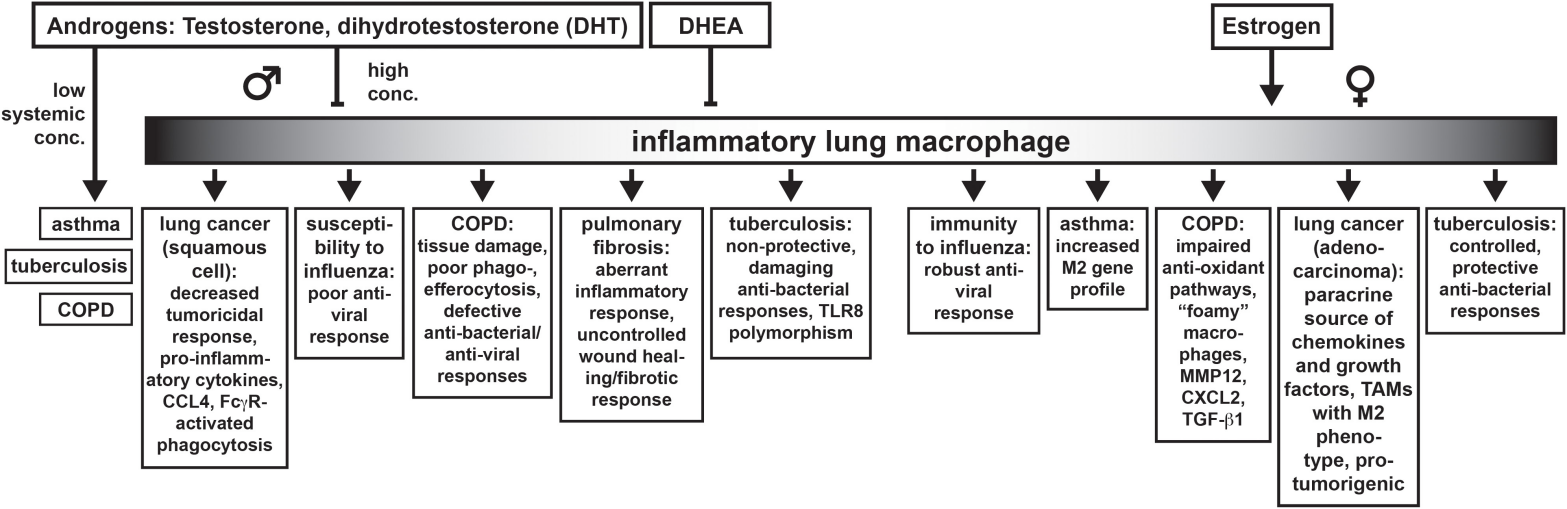
Sex differences in lung diseases discussed in this Review and how they may be connected to the effects of androgens (and estrogens) on inflammatory macrophages in the lung.

In addition to the contribution of structural differences of the lung between the sexes, sex differences in lung function and lung diseases are also dependent on the action of sex hormones. We have summarized some broad concepts that define how testosterone and estrogen affect lung macrophage function and how this may contribute to the outcome of particular lung diseases in Figure 1. As testosterone rises after puberty, the immunosuppressive effects of this hormone on protective immune responses to infectious diseases in males can worsen pulmonary disease. This would be exemplified by tuberculosis or influenza. Some of these effects are a result of androgen effects on critical inflammatory macrophage functions although the effects on the adaptive immune system also have a significant contribution to the overall outcome. Thus, testosterone appears to play a key immunoregulatory role in lung macrophages. Testosterone's immunoregulatory properties also appear to be dependent on the amount of cellular expression of AR and on the concentration of the hormone. Low concentrations of testosterone have been noted in patients with asthma, COPD, and tuberculosis. Low testosterone may also be linked to insufficient control of tissue-damaging inflammatory responses seen in COPD and pulmonary fibrosis. Estrogen tends to promote wound healing responses in macrophages. Dysregulation of wound healing responses and overactive tissue remodeling macrophages in the lung could be broadly used to describe the Th2 response in allergic asthma, which is worse in women. Cancer could also be considered an aberrant wound healing response driven by M2-like tumor associated macrophages. We have highlighted here how sex hormones contribute to changes in lung macrophage function that contribute to lung disease. However, it should be pointed out that not every sex difference in lung disease is due to direct effects on macrophages but on the broader coordinated immune response as a whole.

### Asthma

Before puberty, the structural differences in the lung, as well as gender differences, likely account for the higher incidence of asthma in boys than in girls. With the onset of puberty, male and female sex hormones and their effects on the structural cells of the lung and on the immune system contribute to the incidence of asthma (33, 34). The incidence and severity of asthma are greater in adult women than in adult men (35, 36) and greater in female than in male mice (37, 38). Female sex hormones, such as estrogen, appear to worsen asthma, although a straightforward correlation between amount of female sex hormone and asthma symptoms has not been concluded. Androgens have multiple immunoregulatory and bronchodilatory functions and may contribute to, or be biomarkers for, better lung function in men (39). Accordingly, serum testosterone is low in men with moderate to severe asthma (39–41). In one study, each 25 ng/dL increase in serum testosterone correlated with a 3% (95% CI, 1%-4%; *P* = 0.002) decrease in the likelihood of having asthma (42). On the other hand, high concentrations of testosterone and cyclic AMP in sputum of asthmatic women during the luteal phase of the menstrual cycle were thought to play a role in premenstrual exacerbations (43). The idea that sex hormones may be a causal factor in asthma was significantly strengthened by a recent study of 7,615 adults that quantified serum sex hormones and asthma outcomes (44). That study showed that low testosterone in both women and men was associated with an increased incidence of asthma. The other interesting finding was that higher testosterone was protective against asthma in obese women. Obesity is a risk factor for asthma (45–47). Therefore, how high body mass index (BMI) and circulating sex hormones together affect asthma requires further investigation.

Another androgen, dehydroepiandrosterone (DHEA), also known as androstenolone, is an endogenous steroid hormone and one of the most abundant circulating steroids in humans. It is a precursor for the synthesis of both testosterone and estrogen. DHEA is sulfated at the C3β position into DHEA-S by the action of the sulfotransferase enzymes SULT2A1 and SULT1E1 in the adrenal glands. The amount of DHEA-S in the circulation is ~250–300 times those of DHEA. DHEA became of interest to the asthma field because women with severe asthma had very low concentrations of DHEA-S (48) and DHEA-S concentration correlated with lung function (33). Interestingly, DHEA-S is suppressed by oral or inhaled glucocorticoids, the mainstay therapy for asthma (49). Human DHEA peaks at around age 20 and then follows an age-dependent decline until they reach prepubertal concentrations. Reduced secretion of DHEA with age has been related to a number of age-associated conditions. Replacement of DHEA has been considered as a possible therapeutic that could activate protective responses in an aging immune system. DHEA is known to downregulate Th2-inflammatory cytokines while upregulating IL-2 synthesis (50, 51) in concanavalin A-stimulated peripheral blood mononuclear cells from adult males with atopic dermatitis (52, 53). Thus, it was hypothesized that it would be a useful treatment for atopic diseases including asthma and the results of the clinical trials for DHEA in asthma patients show promise. The results are discussed in a later section titled “*Effects of androgen exposure on monocytes, macrophages in humans with lung disease”*.

### COPD

Sex differences also have been reported in chronic obstructive pulmonary disease (COPD), a heterogeneous, chronic, and progressive respiratory disorder that includes chronic bronchitis and emphysema (54). Chronic exposure of the airways to insults, such as cigarette smoke, leads to epithelial cell injury, destruction of pulmonary capillary vasculature, acceleration of epithelial cell senescence, and airway remodeling. The loss of lung compliance ultimately leads to COPD (55, 56). COPD was previously thought to affect mostly elderly men, primarily because of the higher prevalence of smoking in men. However, as smoking rates increased in women, the number of COPD cases in women exceeded that of men (57). These differences are not only based on gender, as women develop more severe COPD with early-onset disease (<60 years) and have greater susceptibility to COPD with lower tobacco exposure (58). Moreover, increasing age in female smokers leads to a faster annual decline in forced expiratory volume in the first second when compared to that of male smokers, even when they smoke fewer cigarettes (59). Similarly, pulmonary fibrosis is another lung disease that manifests sex differences (60), with men being more affected than women (61, 62). It is characterized by destruction of the pulmonary parenchyma and deposition of extracellular matrix with alterations in phenotype of both fibroblasts and alveolar epithelial cells (63).

### Influenza

The lungs are also the target of respiratory viruses such as influenza A (“flu”), respiratory syncytial virus, and coronaviruses, such as severe acute respiratory syndrome and the Middle East respiratory syndrome. The viruses infect the airway epithelial cells and cause damage to the epithelial barrier by themselves or as a result of the immune response to the viral infection. Sex differences have been noted in the immune response to influenza A virus and to the influenza vaccine. In general, women have a more robust protective immune response to influenza virus and vaccine than do men. Although this elevated response is helpful in clearing virus, women of reproductive age also experience higher mortality and hospitalizations (64–68), possibly from collateral tissue damage to the lungs. The vigorous immune response in women also means that women experience more adverse events after vaccination (69). Indeed, a systems biology approach identified that high testosterone was correlated with a blunted response to the flu vaccine in men (24). As testosterone wanes in elderly men, mortality increases (70). Since the male immune response to the virus is also less robust, the incidence of seasonal flu is generally higher in men than in women in developed countries, according to the World Health Organization (71). It is not yet known how fluctuations in sex hormones across the menstrual cycle and lifespan affect the immune system's response to the influenza virus in humans. Mouse studies have revealed that estrogen is protective at high, but not low, concentrations (72, 73). On the other hand, testosterone replacement in gonadectomized or aged male mice enhanced survival rates (74). Despite these findings in mouse models, studies examining the effect of sex hormones on cellular and molecular mechanisms in human immune cells during influenza infection are lacking.

### Tuberculosis

Like influenza infection, tuberculosis (TB), a lung disease caused by *Mycobacterium tuberculosis*, exhibits notable sex differences in the number of cases worldwide, with men being almost twice as frequently affected than women (75, 76). Both sex and gender differences impact the incidence of TB. Although TB affects less women than men in adulthood (75), women in their economically active years (15–59 years old) have a higher TB incidence compared to women in other age groups (77). This indicates that factors associated with gender, such as exposure to the bacteria, are important in this disease. However, because male predominance does not occur in children, this suggests that biological factors such as male sex hormones also play a significant role (75). This is supported by a study of medically castrated men who experienced a significantly smaller proportion of death from TB, 8.1% compared to 20.6% in intact men (78). Understanding how androgens lead to the greater susceptibility of men to TB is critical, as TB is still one of the leading fatal infectious diseases worldwide and may also may favor the development of other diseases, such as lung cancer (79).

### Lung Cancer

Lung cancer is a very complex disease that depends on a number of variants such as sex, gender, race, and socioeconomic status (80). The development of lung cancer is also related to environmental factors, such as pollution due to industrialization and urbanization (81). An additional gender-associated risk factor, significantly linked to developing lung cancer, is cigarette smoking (80). Historically, more men develop lung cancer and suffer lung cancer-associated deaths compared to women (80). However, the incidence of lung cancer has changed notably in both women and men. In men, lung cancer incidence started to increase in the 1920s and started to decrease in the early 1990s, while in women, the mortality rates and incidence began to rise in the 1960s (80). Changes in smoking habits in the last several decades with a rise in the number of women who smoke correlate with an increase in the incidence of lung cancer in this demographic group (80). Smoking is definitely a key factor in the development of lung cancer; however, recent studies show a higher incidence of lung cancer in young women compared to young men (82, 83), even when the prevalence of cigarette smoking among young women has approached but not exceeded that among men (84). This suggests that the higher incidence of lung cancer in women is not explained simply by gender differences in smoking habits: a deeper analysis of differences mediated by sex, such as greater sensitivity to tobacco smoke in women is warranted (85, 86).

Furthermore, men and women develop different specific types of lung cancer. Malignant mesothelioma is more common in men, while women develop more adenocarcinoma (87), particularly non-small cell lung cancer (NSCLC) (88). Women have a superior survival rate for lung cancer compared to men (89). Tumor-associated macrophages are critical in tumor progression yet how androgens influence macrophage behavior in lung cancer and in responses to treatment must be addressed more deeply to develop better therapies and increase survival rates in men.

## The Myeloid Immune System in Lung Health and Disease

### Alveolar Macrophages

The lungs are a primary interface with the external environment. The delicate structures needed for gas exchange make them susceptible to damage from invading pathogens and toxic molecules. Some insults to the lung can lead to the development of chronic conditions such as allergic asthma. As a protective mechanism, alveolar macrophages clear the air space of infectious, toxic, or allergenic particles to maintain homeostasis in the alveoli. Thus, alveolar macrophages have a dual function as inflammatory cells, phagocytosing and killing inhaled bacteria or viruses, and also as controllers of the inflammatory immune response, minimizing alveolar damage. Resident alveolar macrophages are seeded embryonically from yolk sac and fetal liver monocytes (90–92). In asthma and other lung diseases, recruited alveolar macrophages derived from blood monocytes can turn into pathogenic cells, worsening the condition (93, 94). Mouse alveolar macrophages are characterized by high surface expression of Siglec F and produce TGFβ. TGFβ both supports AM development (95) and their maintenance of immune homeostasis by induction of Tregs and suppression of B and T cell proliferation. Another important function of AM is the clearance of surfactant. AM from male and female mice respond differently to surfactant protein A (SP-A) (96, 97). SP-A acts as an opsonin and is important in clearance of pathogens. Sex differences in AM responses to surfactant could affect bacterial clearance and regulate the production of proinflammatory mediators. The molecular mechanisms that mediate these differences and how sex hormones change this important AM function is an open question.

In the human lung, there appears to be more diversity in the subtypes of lung macrophages compared to mice. The main determinant of the frequency of subtypes of macrophages in humans appears to be their anatomical location within the lung. AM are the predominant immune cells in the lung airways (bronchi and bronchioalveolar space). Flow cytometric panels have employed HLA-DR, CD163, CD169, and CD206 to differentiate between AM, IM and monocytes. Human AM were identified as large, highly autofluorescent CD14- CD16+ cells that also express CD206, CD169, and MARCO (98, 99). There appear to be two populations of AM distinguished by either high or low expression of CD163. More recent approaches to characterize the macrophage populations in the lung involve single-cell transcriptomic analysis (100, 101). Although macrophages show a large variation in the transcriptional phenotype, expression of *MARCO, CCL18, APOC1, APOE, PPARG*, and *MRC1* was found in macrophages from healthy donors (100, 101), while *CHI3L1, MARCKS, IL1RN, PLA2G7, MMP9*, and *SPP1* were highly expressed in macrophages from pulmonary fibrosis patients (101). Thus, a second contributor to diversity is likely the activation state of the cells. There are no data that describe sex differences in human AM responses and the effect of sex hormones on these cells. From our mouse and human MDM studies, we would predict that androgens augment the immune homeostatic functions of these cells in the male lung. Further work is still needed to standardize characterization of the different subpopulations of human lung macrophage populations and their role in maintaining healthy lung function and in disease.

### Interstitial Macrophages

Interstitial macrophages (IMs), are another macrophage population found in the lung. They are a minor population of monocyte-derived macrophages (102), which comprise 30–40% of lung macrophages (103) and are localized in the lung parenchyma (104). IMs contribute to maintaining homeostasis through the spontaneous release of IL-10, a cytokine that dampens inflammation (105). IMs can prevent the development of aberrant type 2 allergic responses triggered by inhaled allergens (104) and have been related to reduction of asthma (106, 107). Different subpopulations of IMs have been found in the lung; however, their characterization has not arrived at a consensus due to difficulties in their identification and isolation. In the mouse lung, different subpopulations of IMs have been described based on the expression of surface markers. One report described three different subpopulations of IMs based on the differential expression of proinflammatory cytokines, chemokine ligands, MHC-II, CD11c, CD206, and Lyve-1 (108); other group identified two subpopulations, based on similar markers but including CX3CR1 (109). Moreover, IMs subpopulations can be also described based on the different anatomic locations these cells populate inside the mouse lung parenchyma (110). Further work is needed to better characterize and define the different IM populations, as the different subtypes may have different functions during the inflammatory process. Smaller in size than their AM counterparts, human IMs express more of the monocytic marker CD14 than AM, perhaps suggesting their monocytic origin, and have lower expression of CD169 than human AM. The responses of IM to androgen will depend on their expression of AR which has not been measured. This will be a challenge due to difficulties in clearly identifying this population (and its subpopulations) from the monocytic, AM and other myeloid populations in the lung.

### Monocytes

Monocytes are produced in the bone marrow along with a number of other myeloid cells. Myeloid cells originate from common pluripotent hematopoietic stem cells and represent the major subset of white cells in circulation (111). These cells comprise basophils, neutrophils, eosinophils, DCs, monocytes, and macrophages, among others (112). Monocytes are released into circulation, then blood monocytes are recruited into inflamed tissue and can mature into macrophages or dendritic cells. There are two main subsets of mouse monocytes, “classical” or Ly6C^high^ monocytes that originate directly from Ly6C^+^ precursors, and “non-classical” or Ly6C^low^ monocytes that derive from Ly6C^high^ monocytes (113). The origin of Ly6C low monocytes was demonstrated by Sunderkotter, et al. by tracking the maturation of DiI-labeled Ly-6C^high^ monocytes into DiI-labeled Ly6C^low^ monocytes (114). This process depends on the transcription factor Nr4a1, which regulates the development and survival of Ly6C^low^ monocytes (113). These two monocyte subsets mirror the human CD14^+^ classical and CD16^+^ non-classical monocyte populations, respectively (115). Ly6C^high^ monocytes highly express the chemokine receptor CC-chemokine receptor 2 (CCR2), whereas Ly6C^low^ monocytes highly express CX3CR1 (116). Importantly, CCR2 expression is required for Ly6C^+^ monocyte egress from the bone marrow into the circulation and entry into non-inflamed and inflamed tissues (117–119) from the blood (120). As monocytes migrate into tissue, they mature into macrophages developing unique, tissue-dependent morphology and functions (121). They lose expression of Ly6C and gain expression of MHC class II, becoming more efficient antigen-presenting cells (122). Some authors have proposed the concept of “tissue monocytes,” which are monocytes that can enter non-lymphoid organs without obligatory differentiation into macrophages. Therefore, monocytes are much more than simply precursors for macrophages.

In human lungs, monocytes, which can be both beneficial and pathogenic in a variety of pulmonary diseases (123), are present at steady state (124). Multiple-color cytometric analysis on cells obtained from different anatomical locations of the lung of healthy subjects (non-smokers with normal lung function and absence of disease or infection) revealed that while intermediate monocytes (CD14^+^CD16^+^) are more frequent in the airways, classical monocytes (CD14^+^CD16^−^) are more frequent in blood (124). Moreover, the different monocyte subsets produced TNF-α to different degrees upon stimulation with TLR ligands (3,4, and 7/8). Thus, the anatomic location where samples are obtained should be considered and reported when working with human bronchoscopies, as this may alter the type and abundance of monocytes and macrophages found. Accurate identification of monocytes in the lung compartments in humans has been a challenge because monocytic “contamination” from the blood vessels (125, 126). Overcoming this challenge, Desch et al. performed a flow cytometric phenotyping study and identified two additional lung monocyte populations by analyzing lungs obtained from donors who died of non-pulmonary causes (127). CD14^+^ CD206^−^ CD1c^−^ CD1a^−^ intravascular monocytes were similar to CD14^+^ blood monocytes and CD14^+^ CD206^+^ CD1c^−^ CD1a^−^ monocytes were described as tissue “monocytes.” These studies highlight that we are just at the beginning of understanding the complexity of lung monocyte subtypes and their functions depending on the inflammatory state of the lung.

Other myeloid populations, like DCs, occupy the lung parenchyma at steady state, and their relative numbers change during inflammation. We refer readers to previous excellent reviews in this journal that cover the importance of DCs in immune responses in the lung and how they are affected by sex differences. Therefore, we will not discuss DCs here (2, 128–132).

### Macrophage Activation

Polarization is a very important effector characteristic observed in monocytes and macrophages. Polarization refers to the change in phenotype and function of monocytes and macrophages as they are exposed to different inflammatory milieus or factors in the tissue microenvironment. To understand the effects of the differing inflammatory or tissue environments on monocyte-macrophage phenotype and function, researchers have used cytokines and other factors *in vitro* to mimic different inflammatory and tissue microenvironments. Monocytes and macrophages stimulated with interferon-γ, LPS, TNFα, interleukin (IL)-12, and granulocyte-macrophage colony-stimulating factor promote a pro-inflammatory macrophage phenotype denoted as M1 polarization. The activation state was also known as “classical” activation. M1-polarized macrophages mediate immunity to intracellular infections, such as viruses and bacteria, and they are generally considered tumoricidal (133–136). M1 macrophages accomplish these functions by inducing production of nitric oxide, reactive nitrogen intermediates, reactive oxygen species, and hydrogen peroxide (137–139). In contrast, activation of macrophages with IL-4 or IL-13, as in extracellular parasitic infections and allergic reactions, leads to M2 polarization or “alternative” activation of macrophages (140). M2 macrophages produce inflammatory mediators and chemokines, such as chitinase-like proteins (141), IL-13 (142), CCL17, CCL18, CCL22, and CCL24, which activate Th2 cells and promote eosinophil infiltration into the lungs (143, 144).

In allergic asthma, a Th2-inflammatory response to inhaled allergens drives lung macrophages toward an M2 phenotype. Increased number and percent of M2 macrophages have been correlated with asthma severity and a decline in lung function in humans and mouse models (145–147). Similarly, M2 macrophages are the predominant subset seen in pulmonary fibrosis and are responsible for fibrogenesis (148). During COPD, the number of macrophages in airways, lung parenchyma, bronchoalveolar lavage fluid, and sputum increases (149, 150). This increase may occur as a result of enhanced monocyte recruitment from circulation in response to chemokines such as CCL2 and CXC-chemokine ligand-1, which are increased in the sputum and bronchoalveolar lavage fluid of patients with COPD (151). Unlike in allergic asthma and pulmonary fibrosis, macrophages in COPD are polarized toward an M1 profile (152). In addition to affecting men and women differently, another commonality of COPD is that macrophages both in the alveolar space and in lung tissue present an altered activation phenotype. Different concentrations of cytokines (TNF-α, IL-1β, IL-6, IL-10, IL-12) and chemokines (CCL2, CCL5, CCL7, CCL13, CCL22, IL-8, CXCL9, and CXCL10) are found comparing smokers to healthy subjects (153–161). Thus the external provoking stimulus uniquely shapes macrophage phenotype and function.

While the M1/M2 designations are useful for *in vitro* studies with stimulation with defined cytokines, the *in vivo* phenotype of macrophages exists on a spectrum somewhere in between these two well-defined opposing phenotypes or does not fit the paradigm at all. For example, M1 and M2 markers can exist simultaneously within the same cell in some cases (162–164). The key factors dictating the macrophage phenotype or activation state are the stage of the immune response and the soluble factors and interactions in a particular tissue microenvironment. For example, the lung environment is rich in GM-CSF, TGFβ, and PPARγ and is critical for development of mature AMs after birth in both mice (90, 91, 165–169) and humans (170–175). Furthermore, interactions between CD200 on type II alveolar epithelial cells and CD200R on the surface of the AM deliver regulatory signals to the AM to prevent proinflammatory signaling and macrophage activation (176). Thus, macrophage nomenclature has evolved as our understanding of the phenotypes and functions of different types of tissue resident macrophages, recruited monocytes and monocyte-derived macrophages advances. In-depth studies of the effects of androgens and other sex hormones on tissue macrophage plasticity and phenotype have yet to be carried out.

## Mechanisms of Androgen Sex Steroid Action

Because androgens are lipophilic steroid hormones, they can easily diffuse across cell membranes without the need for receptor-mediated import (8). Androgens in circulation are found mostly bound to sex hormone-binding globulin and albumin (8). Free (unbound) steroid sex hormones can signal through two different mechanisms: the classical AR, located in the intracellular compartment, and the membrane, or non-classical, AR (8). Androgen binding to classical and non-classical ARs mediates genomic and non-genomic androgen effects, respectively (177). Upon androgen binding, the classical AR undergoes a conformational change and dissociates from heat-shock and other chaperone proteins. An androgen–AR complex is formed that translocates to the nucleus, dimerizes, and binds to androgen responsive elements that modulate the transcription of target genes (178). Importantly, it has been reported that the androgen–AR complex can also mediate non-genomic changes (179) by causing calcium flux and by activating second messenger pathways including ERK, AKT, and MAPK, at least in cell lines (179–181). Whereas, genomic modulation may need hours or days (182), non-genomic modulation can occur within seconds to minutes after androgen exposure, does not involve the complex binding to DNA, and therefore does not affect transcription of target genes (177). DHEA has no known unique receptor and is not a direct AR agonist. It affects immune function but, because it can interact with other sex hormones, it has been difficult to establish its mechanisms of action.

Most studies of androgen–AR complex-mediated gene expression have been carried out in the context of male reproductive tissue in prostate cancer (PCa) (183–185). As previously discussed, immune cells are responsive to sex hormones, and almost all immune cells express sex hormone receptors (8). Mouse monocytes, macrophages (186), and DCs (187) express both classical and non-classical ARs although the vast majority studies do not specifically dissect the role of the two types of AR on the outcomes being measured in the study. Because recent literature has described how sex steroids modulate the functions of DCs (2, 128, 129), we will not discuss it here. We will focus on the importance of androgen–AR regulation of monocyte and macrophage function and how androgen–ARs modulate monocytes and macrophages in lung diseases.

## Androgen Receptor Expression

### Androgen Receptor Expression in Mouse and Human Monocytes and Macrophages

Androgen receptor expression in mouse and human monocytes and macrophages is summarized in Table 1. In general, the expression of the mRNA and protein for classical AR has been assessed, often by non-quantitative means, and non-classical ARs have not been measured.

**Table 1:** Androgen receptor expression in mouse and human monocytes and macrophages.

**Species**	**Cell type**	**Tissue**	**Classical AR**	**Non-classical AR**	**Quantification method used**	**Sex differences**	**References**
Mouse	Macrophage	Bone-marrow derived	✓	✓	Flow cytometry Confocal laser scanning microscopy	M>F	(188) (189)
Mouse	Macrophage	Alveolar	✓		Flow cytometry		(188)
Mouse	Macrophage	Liver	✓			M>F	(190)
Mouse	Macrophage	Adipose tissue	✓		PCR (KO mouse)		(191)
Mouse	Macrophage	Skin	✓		PCR (KO mouse) Immunocytochemistry		(192) (186)
Mouse	Macrophage RAW 264.7 Cell line	Abelson murine leukemia virus-induced tumor; ascites	✓		RT-PCR and western blot		(193)
Mouse	Macrophage	Peritoneal cavity	✓		RT-PCR and Western Blot Protein binding assay		(193) (194)
Mouse	Macrophage Cell line IC-21	Peritonealcavity		✓	Confocal Laser Scanning Microscopy Flow cytometry		(195)
Human	Monocyte derived macrophages	Peripheral blood	✓ ✓		Semi-quantitative PCR Western Blot	M>F	(196) (197)
Human	Monocytic cell line, THP-1 (male)	Peripheral blood	✓ ✓ ✓		Western blot RT-PCR		(198, 199) (193)
Human	Monocyte	Peripheral blood	✓		RT-PCR		(193)
Human	Macrophage	Synovial tissue	✓		Immunohistochemistry; RT-PCR		(200)

## Effects of Androgen Exposure on Monocytes, Macrophages *in vitro*

**Figure 2:**
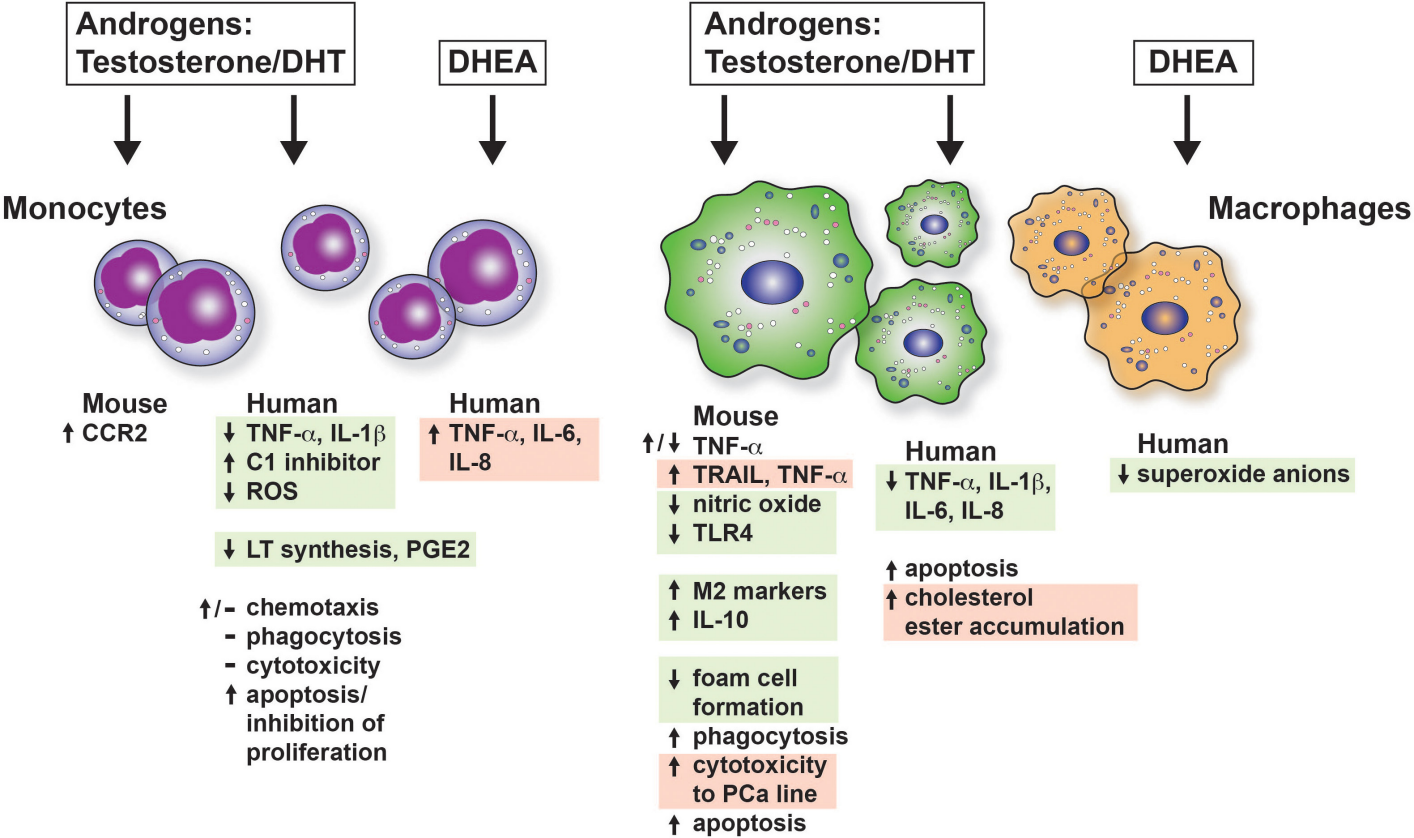
The effects of androgens on mouse and human monocytes and macrophages *in vitro*. The consequences of androgen exposure of monocytes and macrophages are shown. Green boxes indicate that the effect is to dampen proinflammatory responses or be considered immunosuppressive. The red boxes indicate a response that would increase inflammation. CCR2, C-C chemokine receptor type 2; DHT, dihydroxytestosterone; DHEA, dihydroepiandrosterone; PCa, prostate cancer; PGE2, prostaglandin E2; ROS, reactive oxygen species.

We have summarized the outcomes of many studies on mouse and human monocyte-macrophages responses in the presence of androgens in Figure 2. In general, monocyte-macrophage exposure to androgen results in a reduction of pro-inflammatory responses (boxed and shaded in green). It is possible that the reduction in inflammation by androgen may be due to AR suppression of estrogen/ERα-driven pro-inflammatory responses. AR was demonstrated to inhibit ERα activity by binding EREs in breast cancer cells (201). Whether this indirect mechanism accounts for the broad immunosuppressive effects of androgens in normal untransformed immune cells is not known. In keeping with reduced pro-inflammatory responses, we found that androgen enhanced IL-4-induced M2 polarization of bone marrow derived and alveolar macrophages *in vitro* and macrophage-specific deficiency of AR diminished M2 polarization of lung macrophages *in vivo* (188). In some cases, however, inflammatory responses are increased by androgens (boxed and shaded in red). The different responses may be due to different types of tissue macrophages or experimental system. Monocyte-macrophage responses are dependent on the concentration of the hormone, expression of AR, and upon the inducing stimuli to which the macrophage is exposed. The majority of *in vitro* studies examining the effects of androgens on monocytes and macrophages have not clearly acknowledged or separated the effect of androgen on membrane ARs and non-classical AR signaling from that of classical ARs. Therefore, we have to assume that the studies described in the section below are a result of classical AR activity unless explicitly investigated or stated. Determining how non-classical AR signaling and androgen-independent activation of AR affects monocyte and macrophage function is a gap in our knowledge that must be addressed in future studies.

### Effects of Androgen Exposure on Mouse Monocytes and Macrophages *in vitro*

Androgens modulate the expression of proinflammatory molecules such as TNFα in mouse monocytes and macrophages. In 2009, Lai et al. (192) demonstrated that LPS-induced production of TNFα was decreased in BMM lacking classic ARs. Moreover, they found that AR, in the presence of DHT, induced TNF-α promoter activity (192). On the other hand, several reports have suggested the contrary. In one study that used splenic macrophages from midline laparotomy trauma-hemorrhaged mice, DHT suppressed TNF-α production from LPS-stimulated cells (202). This effect was also observed in the mouse macrophage cell line J774 (203), in which testosterone inhibited TNF-α production. In addition, testosterone also decreased expression of the proinflammatory molecule nitric oxide in response to LPS in the mouse macrophage cell lines RAW 264.7 (204) and J774 (203), but it enhanced the expression of IL-10 in the latter.

Other molecules important in monocyte-macrophage functions are also affected by androgens. For example, the expression of CCR2 was enhanced in mouse monocytes by androgens and thereby enhanced chemotaxis (192). However, suppressing AR with siRNA in prostate cells increased macrophage recruitment via CCL2 upregulation, which might promote prostate cancer (205). Phagocytosis was increased by testosterone in rat peritoneal macrophages at 10^−12^ M but not at concentrations lower or higher than 10^−12^ M (206). Cytotoxicity of RAW macrophages to the mouse prostate cancer cell line, TRAMP C2, was enhanced by DHT alone (193). This was attributed to enhanced expression of the M1 polarization markers, TRAIL and TNF-α, in the macrophages. Testosterone (100, 200, and 400 nM) induced apoptosis in mouse BMM through Fas-FasL (207) and activation of caspase 3, 8 and poly (ADP-ribose) polymerase (208).

In terms of M2 polarization of macrophages, we showed recently that *in vitro* exposure of BMM to DHT prior to IL-4 stimulation enhanced *Chi3l3* and *Arg1* gene expression, as well as production of YM1 (188). Androgen amplified the M2 phenotype by increasing IL-4-mediated M2 polarization. Our results were similar to those found in response to IL-4 in the RAW cell line (209). This enhanced M2 macrophage polarization correlated with decreased TLR4 expression and sensitivity to a TLR4-specific ligand observed in testosterone-treated RAW cells (210).

Taken together, these observations suggest that androgens and ARs can either promote or suppress inflammatory properties of mouse macrophages, depending on the external environmental conditions, AR expression, and concentration of hormone. Overall, androgens are more likely to reduce polarization of M1 macrophages. This could represent an important mechanism by which inflammatory pathways are downregulated in males. The opposite effects seen in different inflammatory contexts highlight the need for a deeper and broader study of the androgen/AR-mediated modulation of monocytes and macrophages, as these cells participate in both the initial and late phases of immune responses in a variety of diseases. Most of the studies analyzing the role of AR have focused on prostate cancer, primarily in transformed cell lines (211–213) but macrophages are vital in cancer development and metastasis (205). Furthermore, it is important to consider that opposing effects could result from differential activation of either classical or non-classical (AR-independent) effects (195, 214) which have been rarely studied to date.

### Effects of Androgen Exposure on Human Monocytes and Macrophages *in vitro*

Androgens affect a number of key monocyte and macrophage functions. Studies of androgen receptor function in human monocytes and macrophages have focused primarily on the roles of male sex and sex hormones in promoting atherosclerotic foam cell formation (196) and inhibiting cutaneous wound healing (186, 215). Foam cells are a type of macrophage localized in the blood vessel walls where they engorge cholesterol (216). Foam cells exhibit enhanced inflammatory cytokine secretion and cause atheroma, contributing to cardiovascular disease (216, 217). The effect of androgen on monocytes and macrophages in other immune-mediated human diseases where monocytes and macrophages play a role has been neglected.

The degree of AR expression in monocytes and macrophages is likely the primary determinant of responsiveness, although most studies examining responses to androgens do not quantify AR expression (see Table 1). The expression or action of androgens on non-classical ARs in human monocytes and macrophages has yet to be examined carefully. Most studies assume that the outcomes that are measured are a result of the activity of classical AR. Sex differences in AR content may also play a role in responsiveness. This fact highlights the importance of considering the sex of cells in all *in vitro* studies to accurately assess how sex hormones affect the responses of monocytes and macrophages.

#### Apoptosis, Survival, Proliferation, and Differentiation

Apoptosis was significantly greater in human THP-1 cells cultured for 7 days with 10 nM testosterone than in control cells or cells treated with estradiol (E2), owing to a reduction in proliferating cell nuclear antigen, induction of poly-ADP ribose polymerase-cleaved, an increase in IκB-α, and a decrease in phosphorylated IκB-α (218). E2, in contrast, promoted cell survival. Other studies noted concentration- and time-dependent regulation of apoptosis in THP-1 cells, with an increase in the proto-oncogene Bax and Fas (219). Androgen exposure inhibited proliferation of the human monoblastic leukemia cell line U937, depending on the concentration and time of exposure (220). Cell cycle arrest occurred at the G2/M phase, although another study measured no effect of testosterone on PMA-differentiated U937 cells (221). How testosterone regulates apoptosis and survival of untransformed primary human monocytes and MDMs has not been well-studied. Toxicity was observed when monocytes were differentiated into macrophages over 8 days in the presence of 0.1 mg/mL androgen, but not at lower concentrations of the hormone (222). Testosterone reduced the viability of monocytes from a healthy control and a patient with systemic lupus erythematosus in a concentration-dependent fashion (223, 224). These two studies highlight the importance of concentration in studies of sex hormones. An additional example is the finding that E2 enhances TNF-α secretion from antigen-stimulated T-cells at low concentrations and inhibits secretion at high concentrations (225). IL-1β-induced NF-κB activation is also inhibited at high but not at low E2 concentrations (226). Hence, it is important to carry out *in vitro* studies of sex hormone responses over a wide range of physiologic concentrations of sex hormones.

#### Cytokine Secretion/Reactive Oxygen Species/Inflammation

In general, androgens have a suppressive effect on proinflammatory cytokine expression in monocytes and MDMs. This finding is consistent with the idea that the immune system of females produces cytokines in response to pathogens and insults more robustly than that of males. Monocyte or MDM expression of TNF-α, IL-1β, IL-6, and IL-8 is reduced in the presence of testosterone (227–229). Many studies in this field have relied on human cell lines, such as THP-1 and U937, with or without PMA-induced differentiation into macrophages, and differentiated HL-60 cells, although primary monocytes and MDMs have been used in a few cases (224, 230). Another immunoregulatory function of testosterone is the upregulation and secretion of C1 inhibitor (C1INH) from monocytes (231). C1INH is a 105 kDa plasma protein whose main function is inhibition of the complement system to prevent spontaneous activation. Thus, testosterone keeps complement activation in check. Another mechanism by which testosterone limits inflammation is by decreasing the generation of reactive oxygen species generation from differentiated HL-60 cells. Interestingly, the production of reactive oxygen species in response to zymosan, but not LPS, was inhibited by testosterone (228).

In terms of allergic immune responses, metabolism of arachidonic acid into inflammatory leukotrienes (LTs) via the 5-lipoxygenase (5-LO) pathway is sex-dependent in human monocytes. Pergola et al. (232) reported that primary human peripheral blood monocytes from women synthesize more 5-LO product than do the same cells from men. 5α-DHT (10 nM) suppressed LT synthesis in female cells to the levels observed in males. ERK activation by androgens reduced phospholipase D activity in monocytes and impaired 5-LO product formation by reducing active diacylglycerides. The other branch of arachidonic acid metabolism is the cyclooxygenase (COX) pathway, which generates prostaglandins. Prostaglandin E2 (PGE2), one of the most abundant COX products produced by the airway epithelium and smooth muscle (233, 234), can either stimulate or suppress immune cell function. Testosterone reduced PGE2 production in monocytes obtained from heparinized peripheral blood of healthy adults and incubated for 24 h with LPS (235).

A few studies have examined the effect of DHEA on human monocytes and macrophages. In the presence of LPS, DHEA induced IL-6 and TNF-α production by primary human monocytes and IL-8 and TNF-α production by THP-1 cells (236). In these experiments, DHEA counteracted the effects of cortisol and the glucocorticoid receptor on LPS-induced IL-6 and TNF-α by inducing expression of the scaffolding protein RACK1 (Receptor for Activated C Kinase 1) in THP-1 cells and primary human monocytes (237). RACK1 is involved in multiple signal transduction cascades, including the MAPK, protein kinase C, and Src signaling pathways. RACK1 shuttles proteins around the cell, anchors proteins at particular locations, and is involved in cell migration (238). In contrast, DHEA added to alveolar macrophages lavaged from 11 non-smoking asbestos workers significantly reduced superoxide anion release *in vitro* (239), consistent with its role in dampening Th2-inflammation (240). Therefore, the effect of DHEA on monocytes and macrophages may be stimulus-dependent and needs more in-depth investigation.

#### Foam Cell Formation/Lipid Handling/Atherosclerosis

The formation of foam cells (lipid-filled macrophages) is generally associated with the pathogenesis of cardiovascular diseases, such as atherosclerosis. However, foam cells are also found in patients with silicosis (241) and other fibrotic lung diseases (242) and in tuberculosis. Alveolar macrophages take up extracellular and intracellular lipids in response to inhaled silica, vaping products (243), and *Mycobacterium tuberculosis* (244). Furthermore, the metabolism of fatty acids by macrophages by β-oxidation for sustained energy production is a key feature of the functional phenotype of macrophages with a pro-resolving, tissue reparative (M2) phenotype. Therefore, we have included how androgens modulate foam cell formation and lipid handling in macrophages as part of this discussion.

Macrophages from men and those exposed to testosterone favor the processes of lipid handling and foam cell formation, supporting evidence that atherosclerosis is a male-dominant disease when age is taken into account (245). Atherosclerotic plaques composed of a number of different immune cells form in blood vessel walls. In advanced stages of atherosclerosis, macrophages in plaques take up oxidized low-density lipoprotein (LDL), creating foam cells. Eventually, cholesterol crystals accumulate, trigger inflammation and plaque rupture. The role of sex in the inflammatory events of atherosclerosis has been reviewed elsewhere (246). *In vitro* studies have sought to ascertain how testosterone promotes these processes by utilizing primary MDMs. In MDMs from healthy men, androgen treatment was shown to upregulate genes involved with lipoprotein processing, transporter proteins, cell-surface adhesion, and other pathways, but none of these genes were upregulated in female macrophages (247). The marked sex specificity of androgen effects on human macrophage gene expression is most likely related to sex differences in MDM AR expression. Similarly, treatment of MDMs with modified and native LDL led to changes in expression of mRNAs involved in homeostatic regulation of lipid metabolism, depending on the sex of macrophage donors (248).

Functionally, androgen-treated MDMs from men but not women accumulate cholesteryl esters (196). Male macrophages exhibit increased rates of lysosomal acetylated LDL degradation and upregulated expression of scavenger receptor class B type I (249), increasing high-density lipoprotein (3)-induced cholesterol efflux. The expression of AR in monocytes/macrophages also upregulates lectin-type oxidized LDL receptor 1 molecules that are involved in foam cell formation (198). However, Corcoran et al. (250) observed no effect of testosterone on cholesterol content or efflux from MDMs of healthy male and postmenopausal female donors (age 50–70 years). Because their study used healthy donors, it is possible that the absence of other health-related factors, such as smoking, poor health, and genetic risk factors for coronary heart disease in the healthy blood donors may have produced these results.

#### Migration, Phagocytosis, and Cytotoxicity

Chemotaxis of THP-1 cells was diminished when androgen receptor was knocked down by siRNA suggesting a role for AR in migration of monocytes (198). The authors identified TNF-α as a key AR-regulated molecule important in monocyte migration. In contrast, a handful of studies have tested the effect of testosterone on primary human monocyte phagocytosis and migration, but no effect was found (222, 251–253). Testosterone did not change the cytotoxic capacity of monocytes from male donors (age range 18–40 years) to lyse red cells sensitized with IgG antibodies (254).

## Effects of Androgen Exposure on Monocytes and Macrophages in Mouse Models of Lung Disease

Most studies that have used mouse models to investigate sex differences in lung diseases have focused on the role of estrogen and estrogen receptors (255–257). The importance of androgen and ARs in lung disease has been poorly studied. Earlier studies were directed at modulating monocyte and macrophage functions unconnected to AR function, as 15 years ago it was believed that mouse macrophages did not express classical ARs (189). Nevertheless, recent studies have examined sex differences in mouse models of allergic asthma, COPD, and influenza.

We and others have reported sex differences in mouse models of allergic lung inflammation (37, 38, 188, 255). Some of the observed differences have been clearly attributed to the effect of androgens. We showed that DHT reconstitution of castrated male mice reduced overall lung inflammation (188). A reduction of total serum IgE and total immune cell recruitment to the lungs, specifically eosinophils, revealed the regulatory effect of androgens on several cell types. However, the unexpected enhancement of the production of the canonical M2 macrophage marker involved in eosinophil recruitment (258, 259), YM1, by DHT in alveolar macrophages (188) showed that androgens have a regulatory or an activating effect depending on the cell type. We demonstrated that deletion of classical ARs on monocytes and macrophages (AR^flox^LysMCre mice) resulted in reduced inflammation (less eosinophil recruitment to the alveolar space), along with less mucus production and lung cell infiltrate, despite no differences in serum testosterone level between AR-sufficient and AR^flox^LysMCre mice (188). This finding indicates the importance of androgens as modulators of M2 macrophage polarization and the critical role of these cells in allergic lung inflammation. Other recent studies have shown that testosterone has an anti-inflammatory role in a mouse model of allergic lung inflammation induced by house dust mite but focused on other cell types in lung, such as Th2 (260) and ILC2 cells (261, 262). Similarly, high concentrations of androgens in circulation have been related to a decrease in the expression of TNFα and other proinflammatory cytokines, such as IL-6 and IL-1β, in rodent macrophage cell models and in human monocytes (203, 223, 224, 230, 263). How androgen and ARs impact functions on IMs still needs to be studied. At the time this review was written, no reports on AR expression in IMs were found. However, we hypothesize that as IMs are derived from blood monocytes (102), but once in the tissue they develop an intermediate size and phenotype between monocytes and AM (103, 264), their expression of AR could be somewhere in between. Therefore, androgen and ARs could regulate the functions and activation of these cells. This requires further study, as IMs are a constitutive macrophage population in the lung, and may play a role mediating sex differences in lung diseases.

Mouse models have also shown that sex differences affect COPD. In 2016, Tam et al. (265) reported that smoke-induced COPD is characterized by small airway remodeling in female but not male mice and that ovariectomy before smoke exposure ameliorates the disease. Another study focusing on α-1 antitrypsin deficiency, the leading genetic cause of emphysema, also uncovered a higher susceptibility of female mice for this condition (266). However, these studies did not determine if androgens mediate resistance to COPD, or if the key to the observed sex differences is ovarian sex hormones. Thus, the role that androgens play in COPD and COPD models remains unclear.

Mouse studies that have focused on sex differences in influenza showed that at moderate influenza virus A (IAV) loads, morbidity, mortality, and the associated inflammatory response is greater in female than in male mice, but that mortality is similar at higher loads (72, 267, 268). The role of sex hormones was well-addressed in these studies. High levels of estrogen in estrogen-reconstituted female mice protected against lethal IAV doses (72), whereas the lower estrogen levels in intact females were associated with greater inflammatory responses and increased morbidity after infection. Similar observations were made after progesterone replacement (269). In males, a decrease in androgen levels after castration increased morbidity and pathology upon IAV infection, but replacement of testosterone or DHT reduced morbidity, mortality, and inflammation (72, 74). These findings suggest that although estrogen may be protective or detrimental, depending on concentration, androgens may suppress inflammation in a broader way.

Gonadectomy studies in mice have been used to uncover the role of androgens in TB. Similar to observations in castrated men, castrated male mice that displayed greater pro-inflammatory responses in the lung (more TNF-α, IFN γ, IL-12, iNOS, and IL-17) than intact males. IFN-γ-activated macrophages (M1 macrophages) control of TB infection in both human and mouse (270). Ovary removal in females did not impact susceptibility to TB (271), suggesting that testosterone is responsible for male susceptibility to TB. We previously reported that DHT enhances M2 macrophage polarization through AR (188). Therefore, we speculate that the greater male susceptibility to TB could be at least in part mediated by enhanced M2 responses that are poorly protective and decrease protective proinflammatory macrophage responses. Formal studies to address this idea as well as how androgen effects on other key immune cell players in TB are needed.

How androgens affect monocyte and macrophage biology in lung cancer models in mice has not been well-studied. Monocytes and macrophages are important cellular players in tumorigenesis. Tumor-associated macrophages (TAMs) can be classified into two phenotypes that are either pro-inflammatory and tumoricidal (M1-like) or promote tumor growth and suppress anti-tumor immune responses (M2-like) (272–274). As mentioned previously, sex hormones augment M2 macrophage polarization, thus, play an important role in lung carcinogenesis. The greater overall incidence of lung cancer in men could be explained by an enhanced M2 polarization by androgens (188). On the other hand, estrogen has been shown to induce tumor angiogenesis (275). Estrogen signaling though the cAMP, MAPK, and AKT pathways with the consequent phosphorylation of ERK and EGFR signaling, along with the enhanced expression of c-myc and cyclin D, results in NSCLC cell proliferation (276). Mouse models must therefore address the role of androgens on monocytes and macrophage function in the establishment and progression of lung cancer in male and female animals.

## Effects of Androgen Exposure on Monocytes, Macrophages in Humans With Lung Disease

Few studies have examined the effect of sex hormones on peripheral blood monocytes and lung macrophages from men and women with asthma or the other lung diseases we have discussed here. In women with asthma, dominance of M2 macrophages in airways and lung tissue has been documented (277) and a connection between female sex and female sex hormones surmised. There is a paucity of literature regarding how introducing or depleting exogenous sex hormones (such as in female-to-male transgender individuals receiving testosterone supplementation or women with estrogen blockade) affects the function of blood monocytes and lung macrophages in men and women with asthma. Most studies correlate concentrations of sex hormones with either inflammatory markers, such as cytokines or chemokines in serum and other fluids, or with lung function measurements. We will summarize below the small number of studies in which androgen concentrations were manipulated in humans and the effects on monocyte or macrophage function.

### Testosterone Replacement in Men

Hypogonadism in men refers to a deficiency in testosterone production from the testes that results from testicular, hypothalamic, or pituitary abnormalities. Klinefelter's syndrome in men, which is a result of additional X-chromosomes (e.g., XXY), is the most common cause of hypogonadism. Testosterone replacement therapy is the primary treatment option to restore physiologic testosterone levels, typically in the range of 300 to 800 ng/dL. In general, exogenously administered testosterone has a suppressive effect on the proinflammatory immune response from monocytes. For example, spontaneous production of proinflammatory cytokines (IL-1β, IL-6, and TNFα) *ex vivo* was reduced or completely absent in the monocytes and DCs from men with type-2 diabetes who had partial androgen deficiency and were treated for 12 months with testosterone replacement. This suppression was maintained for 3 more months after testosterone withdrawal (278). Testosterone replacement therapy also is associated with a reduction or complete abrogation of spontaneous *ex vivo* production of inflammatory cytokines by antigen-presenting cells (279). On the other hand, the circulating monocytes from hypogonadal men treated with testosterone replacement therapy exhibited significantly upregulated expression of CD107b at baseline compared to monocytes from healthy controls. This was also seen after stimulation with CpG oligodeoxynucleotides to mimic bacterial DNA exposure (280). Membrane expression of CD107b, also known as lysosome-associated membrane protein (LAMP)2, is indicative of release of lysosome and/or phagolysosome contents into the extracellular medium, a mechanism that may be involved in killing and/or digesting target cells. These data suggest that testosterone increases the inflammatory function of these cells, an effect that would contrast with its typical role as an immunosuppressant.

The immune system of individuals with Klinefelter's syndrome provides unique insight into the genetic contribution of the X-chromosome and that of diminished testosterone to sex differences in different diseases. Men with Klinefelter's syndrome have an increased risk of developing autoimmune diseases, particularly those that are typically female-dominant, such as rheumatoid arthritis and systemic lupus erythematosus (281). As might be predicted due to the negative effect of lower concentration of testosterone on lung function, men with Klinefelter's syndrome are more likely to be diagnosed with pulmonary diseases, such as COPD and pneumonia (282). Asthma is also reported in these individuals (283–285) and it was successfully controlled with long-acting β-agonists and oral testosterone replacement in one case report (283). At the cellular level, however, cytokine production in stimulated whole blood from Klinefelter's men was similar to that of women (286). These data suggest that the effect of the additional X-chromosome was more dominant than the reduction in circulating androgen in Klinefelter's men. In the same study, however, purified monocytes showed the opposite response: cytokine production from the monocytes of healthy and Klinefelter's men was similar and less robust than that from the monocytes of women. This observation led to the opposite conclusion—that androgen plays a more important role in monocyte cytokine production than does chromosomal complement.

### Androgen Excess in Women With Polycystic Ovarian Syndrome (PCOS)

PCOS is a disease characterized by hyperandrogenism, amenorrhea, and polycystic ovaries. The cystic follicles—ovarian theca cells—produce testosterone that causes significant elevations in serum concentrations of testosterone, androstenedione, DHEA, and DHEA-S. In women with PCOS, serum testosterone is in the range of 45–150 ng/dL (2–5 nmol/L) (287), compared with a range of 20–60 ng/dL in healthy, ovulatory women (288). This endocrinopathy is associated with metabolic disorders, such as dyslipidemia, insulin resistance, metabolic syndrome, and cardiovascular complications. Immune function is impaired in women with PCOS, leading to increased secretion of autoantibodies and increased risk of type 1 diabetes, asthma, and thyroid disease (289). Because androgens downregulate the inflammatory responses that contribute to asthma, one might hypothesize that women with PCOS would have less asthma. However, Htet al. (290) found that asthma prevalence was 15.2% in women with PCOS compared to only 10.6% in women without PCOS (*P* = 0.004). Women both with and without PCOS who had asthma tended to have a higher BMI than those without asthma (290). After multivariable analysis, the authors concluded that both PCOS and high BMI were independently associated with asthma (291). It is therefore possible that testosterone contributes to the chronic inflammatory state that accompanies high BMI and that the metabolic dysfunction overpowers the protective effects of testosterone on asthma development. Few cellular and molecular studies have endeavored to uncover mechanisms that explain the association between asthma and PCOS.

At the cellular level, circulating monocytes from women with PCOS expressed the receptor for advanced glycation end-products (RAGE) more strongly than monocytes from healthy control women (292). AGEs are involved in the pathogenesis of a number of chronic lung diseases, ranging from cystic fibrosis to asthma. RAGE can also bind other alarmins, such as the S100A8/A9 heterodimer (calprotectin) or the high-mobility group box (HMGB)1 protein. Both of these ligands have been implicated in the pathogenesis of allergic asthma (291, 293), as they induce cell proliferation or apoptosis, inflammation, collagen synthesis, and cell migration in many different cell types. The concentration of AGE proteins and testosterone correlated positively, even after controlling for BMI and other metabolic function tests (292). Taken together these two studies suggest that monocytes from women with PCOS would be more responsive to RAGE ligands. This heightened responsiveness could promote cellular inflammatory responses that contribute to asthma pathogenesis. Studies are needed to examine how the increased testosterone in women with PCOS affects circulating monocytes and lung macrophages to increase asthma prevalence in this group.

### Testosterone and DHEA Administration in Asthma and COPD

Testosterone has been administered therapeutically for asthma. In an early study, asthmatic women were given testosterone either daily for 5 days over 2 weeks or daily for 3 days over 2 or more weeks. Although the number of participants in the study was small, 88% saw improved symptoms, with 47% reporting no asthma attacks up to 3 months later (294, 295). No studies have examined the effect of exogeneous testosterone administration on blood monocytes or lung macrophages in men and women with asthma. Testosterone deficiency is also present in patients with COPD (296–304). In a clinical study of exercise and testosterone injection in men with COPD, the interventions did not significantly alter pulmonary function or blood gas variables (305). On the other hand, a retrospective study of two large cohorts of men who commenced testosterone replacement therapy within 12 months of a COPD diagnosis showed a 4.2–9.1% decrease in hospitalizations, dependent on age (306). More work is needed to understand how testosterone and its signaling pathways can be harnessed to alleviate lung disease without affecting reproductive systems or having unwanted metabolic effects.

Asthmatic patients have decreased serum concentration of DHEA and DHEA-S (307–309). Therefore, some clinical trials have tested whether DHEA-S supplementation reduces asthma. Men and women with poorly controlled moderate-to-severe asthma were given nebulized DHEA-S for 6 weeks. This treatment led to a statistically significant improvement in the Asthma Control Questionnaire (ACQ) and trends toward better asthma symptom scores and more symptom-free days and nights (310). Oral DHEA for 2 weeks improved lung function in asthmatic women with low DHEA-S < 200 μg/dL (48). However, neither of these clinical studies examined the cellular component of the disease pre- or post-intervention. DHEA and DHEA-S are also lower in patients with COPD than in healthy controls, and COPD leads to pulmonary hypertension (PH). DHEA supplementation improved the 6-min walk test, pulmonary hemodynamics, and the diffusing capacity of the lungs for carbon monoxide of patients with PH-COPD (311, 312). The therapeutic potential of DHEA is currently being investigated in 24 patients with PH in the EDIPHY (Effects of DHEA in Pulmonary Hypertension) trial. However, outcome measures of this trial do not include examination of the immune cells or the effect of DHEA treatment on those cells. Analysis of immune cell function would add important cellular mechanistic insight to these types of trials and help uncover some of the widespread effects of this hormone on the immune system.

## Discussion, Unanswered Questions and Areas of Future Study

Modulation of monocyte and macrophage function mediated by the interaction of androgen and AR has been examined mostly by correlative studies in humans following lifespan changes in sex hormones or using hormonal manipulation in mouse models of lung disease. Most human-based reports are merely descriptive or correlative and do not consider variables such as age, BMI, and phase of the menstrual cycle as key modulators of circulating sex hormone concentrations. Taking these factors into account should be encouraged if we are to gain a better understanding of the impact of sex hormones in health and disease. Analyses of the function of immune cells from male and female healthy controls and patients with lung diseases are needed to unlock how sex hormones alter the biology of the innate and adaptive immune response.

Studying the role of sex hormones as modulators of the immune system is complex because they interact with other hormonal systems and with one another, and because of the nearly ubiquitous expression of sex hormone receptors in most cells of the body. Males and females have all types of sex steroids, although in different circulating concentrations. In humans, changes in the concentration of sex steroids have implications for lung health and may contribute to disease by affecting the function of the immune system. Female sex hormones have been more widely studied as immune system modulators than have androgens. More focus in the future must be directed to how androgens affect the immune system and the interaction between male and female sex steroids in immune function.

Historically, animal models have used only males as study subjects, leaving females aside out of concern for the variability in results introduced by sexually mature adult females with active estrous cycles. As a result, biomedical and preclinical research has neglected to reflect more than 50% of the world's population. This omission had some notable negative consequences: eight of ten drugs withdrawn by the FDA between 1997 and 2000 had significant health risks to women (313). It was not until 2016 that the NIH addressed this oversight with its requirement to include sex as a biological variable in all research studies (314). The practices of using only male animals, not clearly reporting the sex (and age) of the animals used, and mixing male and female results have obscured a proper understanding of how sex and sex hormones influence normal biology and that of disease states. Moreover, many reports comparing sex as a variable lack strict controls on culture conditions *in vitro*, which can alter the results. For example, if investigators fail to appreciate that animal serum or pH indicators, such as phenol red, may act as a source of steroids or sex hormone receptor agonists and do not clearly report their use, the interpretation and reproducibility of the experiments can be diminished. We strongly advocate for the use of hormone-free serum or animal serum replacements (for human cell studies) and use of culture medium that does not contain sex steroid receptor agonists. Moreover, rigorous experimentation should include careful and detailed reporting of cell culture conditions, donor sex and age for cell studies, accurate age and sex in animal work (adherence to ARRIVE guidelines), and separate male and female results.

Here, we have highlighted the importance of sex hormones as modulators of monocytes and macrophages and the important role of these innate immune cells in lung diseases where sex differences are apparent. These cells are part of a larger response that includes the adaptive immune system as well as the structural cells of the lung that are all affected by the action of sex steroids. As such, how innate cells like monocytes and macrophages shape the pulmonary immune response and how they resolve lung inflammation differently in the male and female lung and in the presence of different sex steroids needs intensive study. Uncovering the cellular and molecular mechanisms will be crucial for finding new ways to treat different lung diseases depending on the sex of the patient.

## Author Contributions

MB-D, MS, and NH wrote and revised the manuscript, interpreted the literature, approved and are accountable for all aspects of the final version. All authors contributed to the article and approved the submitted version.

## Conflict of Interest

The authors declare that the research was conducted in the absence of any commercial or financial relationships that could be construed as a potential conflict of interest.
